# Selectively enhanced expression of prophenoloxidase activating enzyme 1 (PPAE1) at a bacteria clearance site in the white shrimp, *Litopenaeus vannamei*

**DOI:** 10.1186/1471-2172-12-70

**Published:** 2011-12-30

**Authors:** In-Kwon Jang, Zhenguo Pang, Jiaping Yu, Su-Kyoung Kim, Hyung-Cheol Seo, Yeong-Rok Cho

**Affiliations:** 1 National Fisheries Research & Development Institute, #707, Eulwang dong, Jung-gu, Incheon 400420, Republic of Korea

## Abstract

**Background:**

The prophenoloxidase-activating (PO activating) system plays an important role in the crustacean innate immunity, particularly in wound healing and pathogen defense. A key member of this system is prophenoloxidase-activating enzyme (PPAE), which is the direct activator of prophenoloxidase (proPO). Despite their importance in crustacean PO activating system, the studies on them remain limited.

**Results:**

Here we report on a PPAE of white shrimp, *Litopenaeus vannamei *(lvPPAE1), which showed 94% similarity to PPAE1 of *Penaeus monodon*. We found that lvPPAE1 in fluid hemocytes was down regulated after challenge by *Vibrio harveyi *but was enhanced when shrimps were exposed to a bacteria-rich environment for long-term. In *vivo *gene silence of lvPPAE1 by RNAi can significantly reduce the phenoloxidase activity (PO) and increase the susceptibility of shrimps to *V. harveyi*. Although lvPPAE1 was down-regulated in fluid hemocytes by *Vibrio *challenge, its expression increased significantly in gill after bacteria injection, which is the primary bacteria-clearance tissue.

**Conclusion:**

Suppressed expression in fluid hemocytes and enhanced expression in gill indicates selectively enhanced expression at the bacterial clearance site. This is a novel feature for PPAE expression. The results will contribute to our understanding of the PO activating system in crustaceans.

## Background

Innate immunity is of great importance to insects and crustaceans because they lack antibodies [1]. Innate immunity involves phagocytosis, encapsulation, hemocyte coagulation and activation of the prophenoloxidase (proPO) or melanization cascade [2,3]. Activation of proPO generates phenoloxidase (PO), which catalyzes the oxygenation of monophenols to *o*-diphenols and the oxidation of *o-*diphenols to the corresponding *o*-quinones [4]. These are reactive intermediates for melanin synthesis and other physiological processes such as cuticle sclerotization, wound healing and pathogen sequestration [5].

In most cases, the proPO cascade is triggered by a small amount of microbe-derived molecules such as lipopolysaccharides (LPSs), β-1, 3-glucans, or peptidoglycan. Pattern-recognition proteins bind these molecules and initiate the proPO system through a quick proteolytic cascade, and many proteins involved in the proteolytic cascade are serine proteinases [6]. The final serine proteinase that converts the inactive proPO into its active form is called prophenoloxidase-activating enzyme (PPAE) [5].

The proPO genes of insects and crustaceans have been intensively studied [7-14], and models for the serine proteinase cascades regulating proPO activation have been studied in detail in some insects such as *Manduca sexta *and *Tenebrio molitor *[15-23]. Studies on proPO-activating cascades are limited in crustaceans. Hitherto, few PPAEs in crustaceans have been reported, except for in crayfish, *Pacifastacus leniusculus *and shrimp, *Penaeus monodon *[24-26]. In this study, the PPAE1 of white shrimp *Litopenaeus vannamei *was identified and its expression feature was reported. The new findings of the study will contribute to our understanding of PO activating system of crustaceans.

## Results

### Isolation and lvPPAE1 sequence

The full-length lvPPAE1 cDNA was 1557 bp, with an ORF of 1389, a 5'-UTR of 57 bp, and a 3'-UTP of 111 bp. The theoretical pI and Mw were 6.96 and 50.4 kDa. The proteolytic activation site was found between Arg228 and Ile229. The pI and Mw of activated lvPPAE1 were 4.5 and 25.4 kDa. The lvPPAE1 protein showed 94% identity to pmPPAE1 and 61% to plPPAE. Three amino acid residues (H270, D319, and S412) corresponded to the catalytic triad of the SP domain and another three amino acid residues (D406, S433, and G435) to the substrate binding sites.

**Figure 1:**
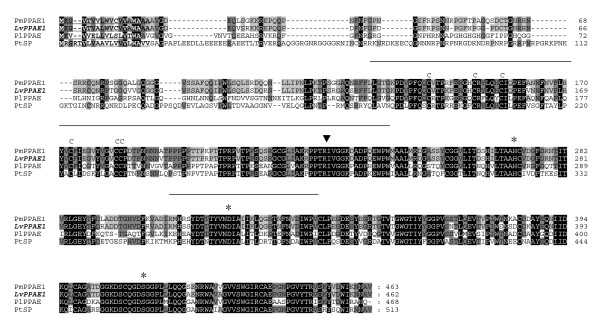
**Alignment of crustacean prophenoloxidase-activating enzymes**. The signal peptides are bold and double-underlined. The six clip-domain cysteines are indicated with the letter "C," and the black triangle indicates the active cleavage site. The amino acid residues corresponding to the serine proteinase catalytic triad are marked by black stars. Glycine-rich and proline-rich domains are marked separately by solid lines. Gene Bank accession numbers are as follows: pmPPAE1 (*Penaeus monodon*, Genebank ID: ACP19558), plPPAE (*Pacifastacus leniusculus*, Genebank ID: CAB63112), ptSP (*Portunus trituberculatus*, Genebank ID: ACI46638).

Multiple alignments between the sequences of lvPPAE1, plPPAE, and pmPPAE1 showed that this gene is relatively well conserved in crustaceans (Figure 1). lvPPAE1 exhibited a typical clip-domain structure, composed of six cysteines at the N-terminus. Similar to other crustacean PPAEs, a glycine-rich domain was found between residues 40 and 130, with 17.6% glycines (16/91, pI = 12.2), and a proline-rich domain was found between residues 194 and 226, with 39.4% prolines (13/33, pI = 12.5).

### lvPPAE phylogenetic analysis

**Figure 2:**
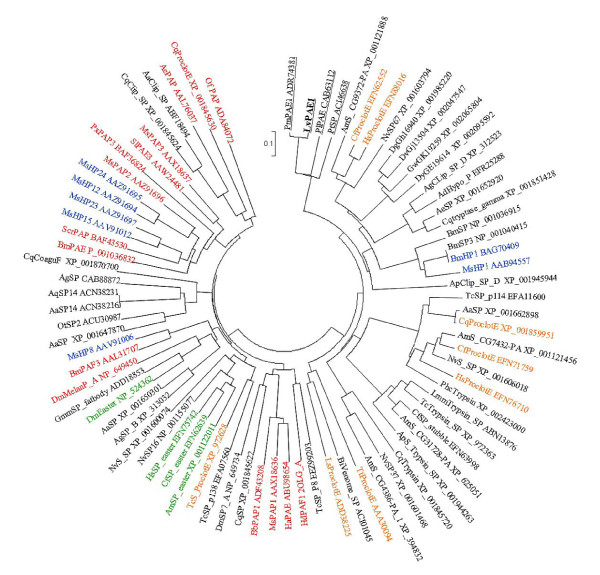
**Neighbor-joining phylogenetic tree constructed with the prophenoloxidase-activating enzyme (lvPPAE1) SP domain and the other 82 clip-domain serine proteinases showing the most similarity in prophenoloxidase-activating proteins in insects**. Bootstrap sampling was reiterated 1000 times. The scale bar refers to a phylogenetic distance of 0.1 amino acid substitutions per site. Prophenoloxidase-activating proteins are highlighted in red, and easter-type serine proteases are in green, hemolymph proteases are in blue, and pro-clotting enzymes are in orange. PPAEs in crustaceans are underlined.

A phylogenetic tree was constructed using the neighbor-joining method (Figure 2) by comparing the deduced amino acid sequence of the conserved SP domain to the 82 clip-domain serine proteinases showing the most similarity or any reported PPAEs. The 83 proteinases were separated into two main branches: one mainly composed of insect prophenoloxidase-activating proteins and the other composed of insect pro-clotting enzymes. The PPAEs of crustaceans were classified into the second main branch and they shared the same and a unique sub-branch. Except for seven pro-clotting enzymes and five trypsins, the biological roles of the clip-domain serine proteinases from the second main branch are still unclear.

### Tissue-specific gene expression in healthy shrimp and bacteria-challenged shrimp

**Figure 3:**
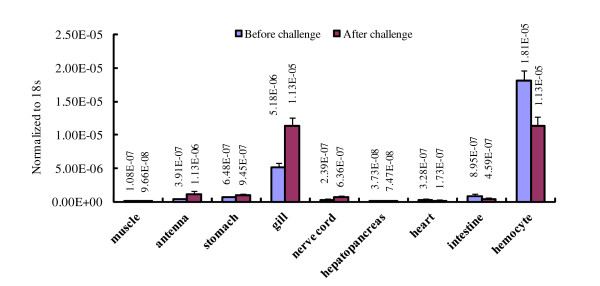
**Tissue distribution of the prophenoloxidase-activating enzyme mRNA from white shrimp *Litopenaeus vannamei *(lvPPAE1) before and after *Vibrio harveyi *challenge**. The transcription level was detected by Taqman qRT-PCR. Gene expression level was normalized to 18 s rRNA. Five samples were taken as replicates.

lvPPAE1 gene expression was analyzed by qRT-PCR. lvPPAE1 in SPF shrimps was mainly expressed in hemocytes and gill, whilst low level was detected in the other tissues. The relative expression in FHs was about 3.5-fold in gill. Forty eight hours after *Vibrio *challenge, the relative expression level of lvPPAE1 in gill was as same as that in FHs, which indicated an increase in the gill (Figure 3). Gill samples from the challenged shrimps were checked using microscope with phase contrast mode. Intensive hemocyte aggregation was found in gill canals (Figure 5A, B).

### Temporal expression of lvPPAE1 corresponding to bacterial challenge

**Figure 4:**
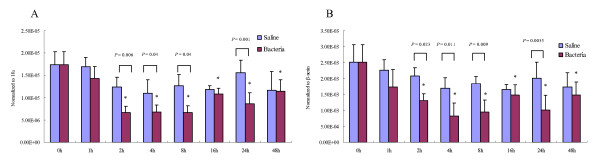
**Time-course mRNA expression of the prophenoloxidase-activating enzyme from white shrimp *Litopenaeus vannamei *(lvPPAE1) in fluid hemocytes after the shrimp was injected with saline or *Vibrio harveyi***. Sampling time points were 0, 1, 2, 4, 8, 16, 24 and 48 h post-injection. Transcript levels were detected by Taqman qRT-PCR. Two sets of data were obtained by normalized to different inferences separately. Five individuals were sampled as replicates. Data were analyzed with *Dunnett's t*-test and *Tukey's *range test. Significant differences from the control (0 h, *P *< 0.05) are indicated by asterisks. Significant differences between challenged and control at each time point are noted by the *P *value above. (A) Normalized to 18 S rRNA. (B) Normalized to β-actin.

**Figure 5:**
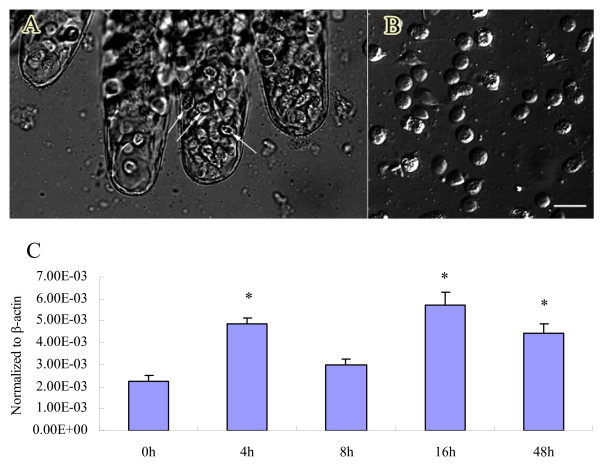
**Hemocyte aggregation and analysis in gills after *Vibrio harveyi *challenge**. (A) Intensive hemocyte aggregation in gill 4 h after bacterial challenge. White arrows show aggregated hemocytes in gill canal. (B) Hemocytes released from gill by dissecting. Bar = 20 μm. (C) Time-course expression of the prophenoloxidase-activating enzyme from white shrimp *Litopenaeus vannamei *(lvPPAE1) in gill after challenge. Sampling times are 0, 4, 8, 16, and 48 h post-infection. Five samples were tested as replicates at each time point. Gene expression level was normalized to β-actin. Data were analyzed with *Dunnett's t*-test. Significant differences from the control (0 h, *P *< 0.05) are indicated with asterisks.

Hemocytes and gill were sampled at each time point after the challenge. qRT-PCR showed that the lvPPAE1 mRNA expression level began to decline in FHs in a short time post challenge. This suppression became more obvious after 4 hpc (hour post challenge); the expression level decreased by 60% compared to the control (0 h). After 16 hpc, the expression recovered slightly but then declined again and remained suppressed after 48 hpc. Multiple comparison between challenged and saline injected groups at each time point showed the suppression of lvPPAE1 were significant at 2, 4, 8 and 24 hpc. Temporal expression in saline injected shrimp was not changed significantly. The two results, normalized to 18 s and β-actin separately, were well matched (Figure 4A, B). However, the relative change in expression in gills was different. At 4 hpc, lvPPAE1 expression in gill was significantly enhanced to about 2.5-fold than that of the control (0 h). After a relative decline at 8 hpc, the expression level increased to 3-fold compared to the control and the high expression was sustained for 48 hpc (Figure 5C).

### Gene expression analysis in shrimp reared in different bacterial environments

**Figure 6:**
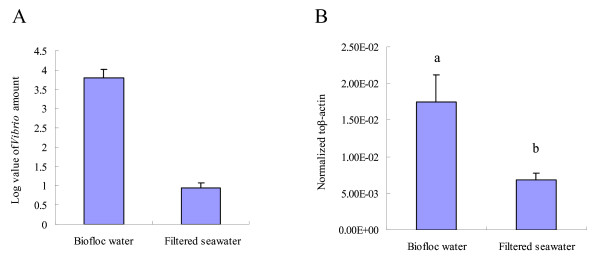
***Vibrio *amounts in different water conditions and corresponding lvPPAE1 mRNA expression levels**. Five samples were taken as replicates. (A) Log value of the *Vibrio *colony amount per milliliter biofloc water and filtered seawater. (B) qRT-PCR analysis of lvPPAE1 mRNA expression level in FHs of shrimp reared in biofloc or filtered seawater. Data were analyzed with the unpaired *t*-test. Different letters indicate significantly different means (*P < 0.05*).

*Vibrio *in the biofloc and filtered seawater was calculated following colony forming unit method after a 24-h culture. The *Vibrio *number in biofloc seawater was 3.5 × 10^4 ^CFU/ml, whereas 45 CFU/ml were found in filtered seawater (Figure 6A). The relative lvPPAE1 mRNA expression level in FHs of shrimp reared in different bacterial environments was analyzed by qRT-PCR. Five samples were taken as replicates for expression analysis. The relative lvPPAE1 mRNA expression level in shrimps exposed to a higher bacteria environment was 2.5-fold than that of those exposed to a lower level of bacteria (Figure 6B).

### RNA interference of lvPPAE1

**Figure 7:**
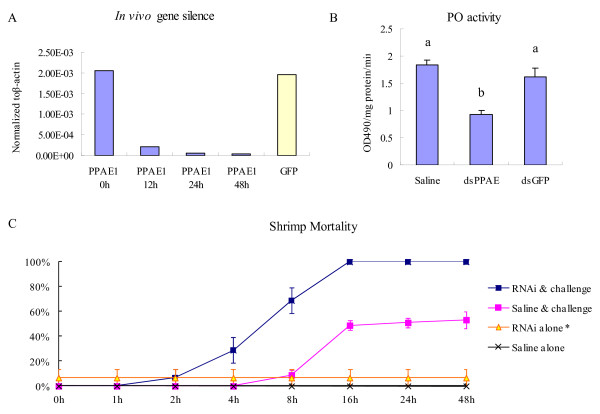
**Gene silencing of the prophenoloxidase-activating enzyme from white shrimp *Litopenaeus vannamei *(lvPPAE1) and related bioassays**. (A) RNAi of lvPPAE1 using gene-specific dsRNA analyzed by qRT-PCR and normalized to β-actin. Five samples were mixed and analyzed 12 and 24 h after the first dsRNA injection and another 24 h after the second injection. (B) Phenoloxidase activity assay after gene silencing, analyzed by analysis of variance (ANOVA) followed by *Duncan's *multiple range test. Five individuals were taken as replicates. Bars with different letters are significantly different (*P < 0.05*). (C) Mortality of shrimps due to *Vibrio harveyi *after gene silencing. The experiment was conducted three times.*Occasional mortality occurred after the first lvPPAE1 dsRNA infection.

qRT-PCR was used to detect the dsRNA interference effect 12 and 24 h after the first dsRNA injection and 24 h after the second injection. Total RNA extracted from the hemocytes of gene-silenced shrimp was analyzed. RNAi strongly inhibited lvPPAE1 expression. The amount of mRNA decreased to less than 5% of the control 24 h after the second lvPPAE1 dsRNA injection (0 h, Figure 7A).

### Susceptibility of shrimp to *Vibrio harveyi *after gene silencing

Susceptibility of shrimps to *V. harveyi *was tested after gene silence. About 1 h after the challenge, shrimps in the gene-silenced group began to show symptoms of slowed mobility and imbalance, as well as mortality after 2 h. Saline-injected shrimp showed symptoms after at least 4 h, and mortality began after 8 h. Mortality in the saline pre-injected group reached about 50% after 48 h, and all gene-silenced shrimp died within 24 h. No mortality was found in the saline-only injected group and occasional mortality was found in the lvPPAE1 dsRNA-only injected group (Figure 7B).

### PO activity assay

PO activity was analyzed in shrimp injected with gene-specific dsRNA, GFP dsRNA, or saline solution. PO-induced activity in the gene-silenced shrimp decreased by approximately 50% compared to the saline-injected control group. No such decrease was found in the GFP dsRNA-injected shrimp (Figure 7C).

## Discussion

We identified and characterized a clip-domain serine proteinase from the white shrimp, *L. vannamei*. The deduced amino acid of this gene showed high similarity to that of plPPAE (61%) and particularly high similarity to pmPPAE1 (94%), which is the terminal enzyme in the prophenoloxidase-activating cascade [25,26]. Thus, we named this gene lvPPAE1. Multiple alignment results indicated that this gene is rather well conserved in crustaceans. Interestingly, phylogenetic analyses revealed that all PPAEs in crustaceans branched off into the cluster of insect pro-clotting proteinases rather than insect PPAEs. Besides with molecular structure deviation, PPAEs in crustaceans can proteolyses proPO dependently [27], unlike most of insect PPAEs that need cofactors to active proPOs [6]. Another major difference between PPAEs in crustaceans and insects is the different regulation type to pathogen invasion. Three prophenoloxidase activating proteinase of the horn worm *Manduca sexta *were reported to be stimulated or up-regulated by injection of *Micrococcus lysodeikticus *or *Escherichia coli *[17,28,29]. Five putative PPAEs of the fly *Drosophila *were induced to higher levels after *E. coli *and *Micrococcus luteus *infection [30]. Two potential PPAEs of the mosquito *Anopheles gambiae *were up-regulated by bacteria *E. coli *or *M. luteus *and parasite *Plasmodium berghei *[31]. A prophenoloxidase activation proteinase of the silk worm *Samia cynthia ricini *was inducible by cell wall components of gram-positive bacteria [32]. Contrary to those insect PPAEs, The crayfish PPAE transcript level was reported to be unaffected upon *Aeromonas hydrophila *challenge [33]. The pmPPAE1 transcript level in *P. monodon *was down regulated 3-24 h after *Vibrio *injection [25]. In the present study, the lvPPAE1 transcript abundance was reduced within 48 h after *Vibrio *challenge. All reported crustacean PPAEs as well as our study showed their transcript levels were either not affected or down-regulated within 48 h, contrary to PPAEs in insects that were up-regulated or stimulated within 24 h after challenge. Dissimilarity in proteinase structures and functional features imply certain degree of difference exists between crustaceans and insects for the prophenoloxidase-activating mechanism, particularly in the down stream.

It has been reported that the PPAE1 mRNA of crustaceans were found specially in hemocyte and free from other tissues [25]. Our tissue distribution analysis by qRT-PCR also revealed that lvPPAE1 transcript level was very low in muscle, antenna gland, nerve cord and digesting system, and this minimum expression also could be caused by a few amount of hemocytes penetrated into the tissues [5]. The most convincing proof was that the lvPPAE1 transcript level in those tissues didn't change after bacteria challenge, suggesting that these tissues were in fact free of lvPPAE1 transcripts. This conclusion is in accordance with the previous report [25]. However, it is notable that during bacteria clearance process in shrimps, bacteria or foreign materials would be encapsulated by hemocytes to form hemocyte-foreign material aggregation and this kind of aggregation happens mainly in gill, which is the main bacteria or alien materials clearance site [34]. This phenomenon was also confirmed by our microscope observation (Figure 5A, B). In another word, gill is closely related with hemocytic immunity in host defense of shrimps. By sensitive qRT-PCR method, we successfully found out the inevitable repercussion of this close relation: The lvPPAE1 mRNA transcripts represented a considerable level and even higher after *Vibrio *challenge.

Our RNAi experiment showed silence of lvPPAE1 significantly reduced the PO activity and increased the susceptibility to *V. harveyi*. qRT-PCR revealed shrimps exposed in a bacteria rich environment for a long-term presented a higher lvPPAE1 transcript level. Those results suggested the importance of lvPPAE1 in the host defense of *L. vannamei*. It is thus odd to find the expression of lvPPAE1 was depressed when shrimps tried to fight against the injected bacteria. Same phenomenon was found in another shrimp species *P. monodon*, yet the author didn't give an explanation. Our findings in gill gave a clue to answer the doubtful question. Although the transcript expression of lvPPAE1 in gill was in fact due to the fixed hemocytes (GH), the transcript level of GHs showed different regulation type than in FHs. Contrary to the continuous dropping trend in FHs, the transcript level of lvPPAE1 in gill was stimulated quickly and arrived to a peak at 4 h when the depression in FHs was at the upmost degree. Higher lvPPAE1 mRNA transcript level was maintained in gill within 48 h when the lvPPAE1 was continuously depressed in FHs. By now, a new conclusion was drawn that the lvPPAE1 was not simply down-regulated by *Vibrio *challenge but also up-regulated in bacteria clearance site. By the current data, it can not be asserted that the overall lvPPAE1 transcript level was depressed or enhanced shortly after the *Vibrio *challenge; we thus call the expression feature as the "selective enhanced expression". Because the selective enhanced expression in gill happens simultaneously with the suppression in fluid hemocytes, the endogenous regulation mechanism for the short time enhanced expression in bacteria clearance site is probably different from the enhanced expression in FHs under a long-term, bacteria-rich environment stress. Our current study on lvPPAE1 indicated that the endogenous regulation mechanism for shrimp immunity is far from clarified.

## Conclusion

A prophenoloxidase activating enzyme (lvPPAE1) of the white shrimp *L. vannamei *was cloned and sequenced. *In vivo *gene silence of lvPPAE1 significantly deduced the PO activity and increased the susceptibility of shrimps to gram negative bacteria *V. harveyi*. The overall tissue distribution analysis showed the lvPPAE1 was mainly expressed in fluid hemocytes (FH) and gill due to the aggregated hemocytes in canal (GH). The lvPPAE1 expression was significantly enhanced in gill within 2 h after *V. harveyi *challenge. In the case of FH, lvPPAE1 expression was suppressed within 48 h after a single challenge, whereas higher expression was found when shrimp was exposed to a high bacterial environment over the long term. The different gene expression responses to bacterial challenge in FH and gill suggest enhanced lvPPAE1 expression at the bacteria-clearance site.

In conclusion, the lvPPAE1 is involved in melanization cascade and plays an important role in shrimp host defense against bacteria. The expression in FH was suppressed in a short term after *V. harveyi *challenge but was up regulated under bacterial press for a long term. The lvPPAE1 represents a feature of selective enhanced expression in bacteria clearance site after challenge. This is a novel characteristic for crustacean PPAEs expression.

## Methods

### Animals, sample preparation and RNA preparation

Specific pathogen-free (SPF) *L. vannamei *(10 g ± 1.2 g mean ± SD) were obtained from a biofloc water culture system at the NFRDI (Seoul, Korea) and were maintained in filtered running seawater for two weeks, if needed. All tissue preparations were performed as bellow: Tissues such as muscle, stomach, antenna gland, nerve cord, intestine, hepatopancreas, or gill were obtained by dissection. Hemolymph was collected from the ventral hemolymph sinus of *L. vannamei *with a 3 ml RNase-free syringe containing on ice pre-cooled anticoagulant (113 mM glucose, 27.2 mM sodium citrate, 2.8 mM citric acid, and 71.9 mM NaCl). The hemocyte cell pellet was collected by centrifugation at 700 × g and 4°C for 10 min followed by two rinses with anticoagulant. All tissues were placed immediately in 200 μl RNA later reagents after preparation (Ambion, Austin, TX, USA). Total RNA was extracted with the RNeasy Mini Kit (Qiagen, Valencia, CA, USA) and further purified with DNase I (Qiagen), according to the manufacturer's protocol.

### cDNA synthesis and rapid amplification of cDNA ends

The first-strand cDNA for 5' rapid amplification of cDNA ends (RACE) and 3' RACE was synthesized separately with the SMARTer™ RACE cDNA Amplification Kit (Clontech, Mountain View, CA, USA), according to the user's manual. The first-strand cDNA was diluted 20 times to serve as the RACE template. A degenerate of the gene-specific primer lvPPAERACE3' was designed based on other crustacean PPAE sequences [25,26]. PCR amplification was performed with the specific primer and two universal primer provided by the kit. The touchdown PCR conditions were as follows: five cycles of 94°C for 30 s and 72°C for 3 min; five cycles of 94°C for 30 s, 70°C for 30 s, and 72°C for 3 min; 25 cycles of 94°C for 30 s, 68°C for 30 s, and 72°C for 3 min. PCR products were then subcloned into the TOPO vector (Invitrogen, Carlsbad, CA, USA) and sequenced. The fragment sequence was confirmed by the BLAST program on NCBI by comparison with other shrimp serine proteases. A new primer, lvPPAERACE5', was designed based on the partial sequence of lvPPAE1. 5' RACE was performed with the new primer and universal primers mentioned above with the same PCR conditions as 3' RACE. Then the 5' RACE product was subcloned into the TOPO vector and sequenced. After the 5' RACE sequence was confirmed by BLAST, 3' RACE fragment and 5' RACE fragment sequences were joined using the Mega 4 program [35].

### Sequence analysis

The open reading frame (ORF) of the gene was analyzed by the ORF finder program on NCBI. The nucleotide sequence and deduced amino acid sequences were analyzed by the BLAST program on NCBI. Multiple alignments were performed with the Clustal W2 program http://www.ebi.ac.uk/Tools/clustalw2/, and the result was viewed with Genedoc 2.7.0 [36]. A neighbor-joining phylogenetic tree was constructed based on the SP domain of the deduced amino acid sequences of lvPPAE1 and the 82 clip-domain serine proteinases showing highest similarity or the prophenoloxidase-activating proteins of insects using the Mega 4 program [35]. The lvPPAE1 signal peptide was predicted with the SignalP 3.0 server http://www.cbs.dtu.dk/services/SignalP/. The theoretical isoelectric point (pI) and molecular weight (Mw) of the proteins were estimated online http://isoelectric.ovh.org/.

### Quantitative real-time PCR for mRNA detection

A Taqman probe-based quantitative reverse transcription PCR (qRT-PCR) technique was taken to test the lvPPAE1 transcript level. Tissue distribution or mRNA expression under different circumstance stresses can thus be evaluated by this method. A pair of gene-specific primers qRTPPAEf, qRTPPAEr and a Taqman qRTPPAEp probe were designed from the full-length lvPPAE1 cDNA sequence using PrimerExpress software (Applied Biosystems Pty Ltd., Melbourne, Australia). One-step qRT-PCR was accomplished with the One Step PrimeScript™ RT-PCR perfect real time Kit (Takara Bio). The reaction mixture consisted of 10 μl 2× One-Step RT-PCR Buffer III, 2 units of Takara Ex Hot Start Taq enzyme, 0.4 μl reverse transcript enzyme Mix II, and 0.4 μM each of forward primer, reverse primer, and Taqman probe in a final reaction volume of 20 μl. The PCR conditions were as follows: 42°C for 5 min, 95°C for 10 s, followed by 40 cycles of 95°C for 5 s and 60°C for 30 s. Fluorescent signal detection was started from the first cycle of the annealing stage. The 18 s ribosomal RNA (18 s) and β-actin was taken as references with primers and probe shown in Table 1.

**Table 1:** Primers and probes used in the experiments

Name	Nucleotide sequence	Note
dsEGFPf	5'-GAATTAATACGACTCACTATAGGGAGACGTGACCACCCTGACCTA-3'	RNAi control forward primer
dsEGFPr	5'-GAATTAATACGACTCACTATAGGGAGAATGCCGTTCTTCTGCTTG-3'	RNAi control reverse primer
lvPPAERACE3'	5'-AGTCACATYCTYACKGCYGCSCACTG-3'	3' RACE primer
lvPPAERACE5'	5'-GGTCGAAGCCGTCAACGCAGTGC-3'	5' RACE primer
dslvPPAEf	5'-GAATTAATACGACTCACTATAGGGAGACTTCCGTCCTTCCAACAAT-3'	dsRNA production forward primer
dslvPPAEr	5'-GAATTAATACGACTCACTATAGGGAGAGCCTCTTGGCGATCAGTC-3'	dsRNA production forward primer
qRTPPAEf	5'-AGTTCCTACGACACGACCACCTA-3'	qRT-PCR PPAE1 forward primer
qRTPPAEr	5'-TCGACGTTGAAGTTGGTGCTT-3'	qRT-PCR PPAE1 reverse primer
qRTPPAEp	5'-AACGACATCGCCATCATCAAGCTGC-3'	qRT-PCR probe
qRTβf	5'-CGAGGTATCCTCACCCTGAAAT-3'	qRT-PCR β-actin forward primer
qRTβr	5'-GTGATGCCAGATCTTCTCCATGT-3'	qRT-PCR β-actin reverse primer
qRTβp	5'-CGAGCACGGCATCGTCACCAA-3'	qRT-PCR β-actin probe
qRT18sf	5'-TGCTCAGAGCAGGCTGGTTT-3'	qRT-PCR 18s forward primer
qRT18sr	5'-GAGGTCCTGTTCCAATCATTCCA-3'	qRT-PCR 18s reverse primer
qRT18sp	5'-TGCTTACAGCCCGAATGGTCGTGC-3'	qRT-PCR 18s probe

### *Vibrio *challenge for temporal expression assay

*V. harveyi *KCCM 40866 obtained from the Korean Culture Center of Microorganism (KCCM) was used in a preliminary challenge trial to determine its pathogenicity. Then it was further selectively enriched from the hepatopancreas of freshly killed shrimp in the preliminary challenge on tryptic soy agar (Difco, Franklin Lakes, NJ, USA) plate. Harvested bacteria were diluted with physiological saline (154 mM) to an OD_540 _of 0.09 (10^6 ^CFU). The diluted bacteria (100 μl) were injected into the third abdominal segment. SPF shrimps (about 10 gram) from biofloc culture system were raised in filtered sea water for 2 weeks before challenge. One hundred and twenty individuals were challenged and a control group of 50 individuals were injected with the same volume of saline. Gill and hemocyte were sampled at 0 h, 1 h, 2 h, 4 h, 8 h, 12 h and 48 h after bacteria or saline injection. Five shrimps for each group were sampled as replicates at each time point separately. Temporal expression was detected by qRT-PCR and the relative expression data were analyzed by *Dunnett's t*- test and *Tukey's *range test.

### *Vibrio *count in biofloc and filtered sea water and corresponding lvPPAE1 mRNA expression

Water samples (200 μl) from biofloc water or filtered sea water were spread on 0.2% sodium-chloride thiosulfate citrate bile salt agar (Difco) and incubated at 28°C for 24 h before the colonies were counted. *Vibrio *colony numbers were counted three times everyday continuously for 4 days. Fluid hemocytes were extracted from SPF shrimps (about 10 gram) raised in biofloc water culture system or filtered running seawater and the lvPPAE1 mRNA level were measured and compared with qRT-PCR. Five shrimps were sampled as replicates from the two culture systems separately. The deference of PO activity was analyzed by unpaired *t*-test for unequal variance.

### dsRNA production

First-strand cDNA was synthesized with the PrimeScript™ First-Strand cDNA Synthesis Kit (Takara Bio) with the oligo dT Primer, according to the manufacturer's protocol. A pair of forward and reverse primers with a T7 promoter oligo anchor (dslvPPAEf, dslvPPAEr) amplifying a fragment of 542 lvPPAE1 nuclei fragments was designed. The PCR amplification was conducted with a reaction mixture of 2 μl 10× PCR buffer, 1.6 μl dNTP mixture, and 0.1 μl ExTaq enzyme with a final primer concentration of 0.4 μM. One μl first-strand cDNA was added to make a final volume of 20 μl with PCR water. The PCR conditions were as follows: 35 cycles of 94°C for 30 s, 59°C for 30 s, and 72°C for 1 min 30 s. PCR fragments were purified by agarose gel electrophoresis and subcloned into the TOPO vector (Invitrogen). Sequences were confirmed by sequence alignment and then used as the template for a secondary PCR with T7 promoter primers only. The secondary PCR product was then used as a template to produce dsRNA. dsRNA was produced and purified using the RiboMAX™ T7 Express System (Promega, Madison, WI, USA), according to the user's manual. dsRNA concentration was calculated by measuring the absorbance at 260 nm with a spectrophotometer (Cary 100 scan spectrophotometer, Varian). An alien GFP protein sequence was amplified with the PEGFP-1 (Clontech) reconstructed vector, using the dsGFPf forward primer and the dsGFPr reverse primer (encoding 303 nuclei), and the produced dsRNA fragment was used as a control.

### *In vivo *gene silencing

lvPPAE1 and GFP protein dsRNA were diluted in 0.9% saline separately. Diluted dsRNA was injected into the third abdominal segment using a 1 ml sterile hypodermic syringe with a 26 gauge needle. Approximately 1 μg lvPPAE1 or GFP dsRNA was used per 1 g shrimp. Another injection with the same amount of dsRNA was performed 24 h later. The gene silencing effect was tested by qRT-PCR.

### Analysis of susceptibility to *V. harveyi*

Susceptibility of lvPPAE1 gene silenced or control shrimp to *V. harveyi *was evaluated. SPF shrimps from biofloc system were pre-raised in 26°C filtered sea water for two weeks and used for experiment. Shrimps were challenged simultaneously with the second dsRNA injection for gene silencing. Mortality of gene silenced or control shrimp was monitored within 48 h. Two additional groups injected with only saline or only lvPPAE1 dsRNA were set as controls to determine mortality from the procedure. Thirty individuals for each group were used to calculate the mortality rate and the experiment were repeated for three times.

### Phenoloxidase activity assay

Hemolymph of shrimps from the gene silenced or control group were extracted with anticoagulant buffer, centrifuged at 700 × g and 4°C for 15 min. The cell pellet was rinsed twice with anticoagulant buffer, resuspended, and centrifuged at 16000 × g and 4°C for 5 min. An aliquot (500 μl) of ice-cold homogenizer buffer containing 10 mM sodium cacodylate, 5 mM CaCl_2_, pH 7.0 (Sigma, Munich, Germany) was added, and the cell pellet was homogenized for 2 min at full speed to release the proteins. The cell homogenate was then centrifuged at 10000 × g and 4°C for 10 min, and the supernatant (LPS) was used for the PO activity test. LPS (100 μl) and 50 μl laminarin (0.1 mg/ml, Sigma) was mixed in a 96-well ELISA plate and incubated at 27°C for 10 min. L-3, 4-dihydroxyphenylalanine (50 μl, 3 mg/ml) was added, and PO activity was measured with an ELISA reader, using dynamic read style, for 20 min at room temperature. Absorbance at 490 nm was measured from 10 to 20 min. Protein concentration was measured with a BCA™ Protein Assay Kit (Pierce, Rockford, IL, USA). PO activity was calculated as OD490/mg LPS protein/min. Data was analyzed by analysis of variance followed with *Duncan's *multiple range test. Five individuals were sampled as replicates.

## Authors' contributions

IKJ designed the study and gave important comments to the MS. ZGP performed the statistical analysis and wrote the MS. ZGP and JPY carried out sequencing, sequence alignment, qRT-PCR analysis, RNAi and challenge experiment and PO assay. SKK participated in PO assay and *Vibrio *counting. HCS and YRC prepared materials and the field facility. They also donated animals for the study.
